# Animal Models of Restenosis and Intimal Hyperplasia in Cardiovascular Percutaneous Interventions: A Narrative Review

**DOI:** 10.3390/biomedicines14020309

**Published:** 2026-01-29

**Authors:** Sabrina Houthoofd, Marc Vuylsteke, Serge Mordon, Inge Fourneau

**Affiliations:** 1 Department of Vascular Surgery, University Hospitals Leuven, Herestraat 49, 3000 Leuven, Belgium; inge.fourneau@uzleuven.be; 2 Department of Vascular Surgery, Sint-Andriesziekenhuis, Bruggestraat 84, 8700 Tielt, Belgium; marc.vuylsteke@mac.com; 3 Hemerion Therapeutics, 37, Rue Denis Papin, 59650 Villeneuve d’Ascq, France; srm@hemerion.com

**Keywords:** restenosis, intimal hyperplasia, animal models, cardiovascular interventions, translational potential, 3Rs

## Abstract

**Background**: Restenosis and intimal hyperplasia following arterial bypass surgery or percutaneous interventions remain major clinical challenges that significantly impair long-term vessel patency and clinical outcomes, despite substantial technological progress. Preclinical research aimed at understanding the biological mechanisms underlying restenosis and developing effective therapeutic strategies relies heavily on experimental animal models. **Methods**: A narrative review of the literature was conducted using PubMed, Embase, Web of Science Core Collection, and the Cochrane Library to identify relevant studies describing animal models of restenosis and intimal hyperplasia following percutaneous cardiovascular interventions. **Results**: The reviewed studies describe a broad range of animal models, including rodents, rabbits, swine, and other large animals, with each species exhibiting distinct anatomical, physiological, and pathological characteristics that influence its suitability for studying restenosis and intimal hyperplasia. Considerable interspecies variability exists in vascular healing responses, inflammatory processes, and translational relevance. **Conclusions**: Animal models remain indispensable tools for investigating restenosis and intimal hyperplasia and for evaluating novel pharmacological and device-based therapies. Understanding interspecies differences is essential for designing appropriate experimental studies and interpreting findings. Careful animal model selection is critical to improving translational relevance and facilitating successful clinical translation.

## 1. Introduction

Arterial bypass surgery and percutaneous endovascular interventions, including balloon angioplasty and stent implantation, are widely used therapeutic approaches for treating coronary and peripheral arterial disease. These procedures have substantially reduced morbidity and mortality associated with atherosclerotic disease. Nevertheless, restenosis remains a significant limitation, occurring in up to 30–35% of cases, depending on lesion characteristics, patient comorbidities, and the type of intervention performed [1,2,3].

**Figure 1:**
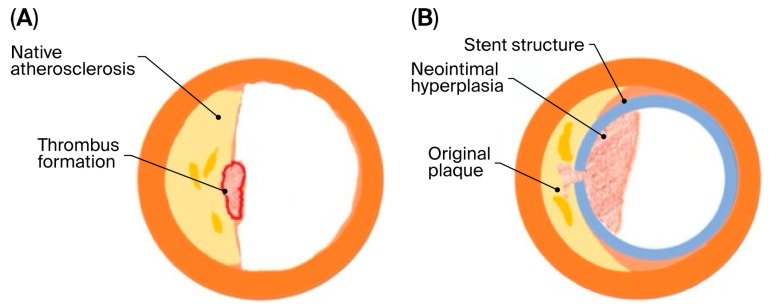
Schematic representation of native atherosclerosis and stent-induced restenosis. (**A**) Cross-sectional view of a native atherosclerotic artery illustrating eccentric plaque formation (yellow) within the vessel wall (orange), resulting in luminal narrowing (white), with superimposed thrombus formation (red) following plaque disruption. (**B**) Cross-sectional view of a stented artery showing the metallic stent structure (blue) and subsequent neointimal hyperplasia (pink) leading to restenosis. The original atherosclerotic plaque (yellow) remains within the vessel wall (orange), while excessive neointimal tissue growth reduces the luminal area (white). Adapted from Nusca et al., *Life* **2022**, *12*, 393, under CC BY 4.0 [7].

Restenosis represents a maladaptive vascular healing response to mechanical injury. Although vessel wall repair is necessary to restore endothelial integrity, excessive or dysregulated healing leads to intimal hyperplasia and adverse vascular remodeling, ultimately resulting in luminal narrowing and impaired blood flow (Figure 1). The biological mechanisms underlying restenosis are complex and multifactorial, involving endothelial dysfunction, platelet activation, inflammatory cell recruitment, smooth muscle cell (SMC) migration and proliferation, and extracellular matrix deposition [4,5,6].

Despite advances such as drug-eluting stents, drug-coated balloons, and improved procedural techniques, restenosis has not been eliminated. This persistent clinical challenge underscores the need for continued investigation into its pathophysiology and the development of innovative therapeutic strategies. Experimental animal models have played a central role in elucidating the key mechanisms of vascular injury and repair and in evaluating potential therapies prior to clinical application.

Since the 3Rs concept (Refinement, Reduction, Replacement) was first introduced by Russell and Burch in 1959, considerable efforts have been made to ensure animal welfare [8]. While replacement is a key principle in the search for animal experiment alternatives, atherosclerosis is a complex, multifactorial disease that cannot be fully replicated in vitro; its pathogenesis is influenced by interactions among multiple cell types, blood flow and shear stress, hyperlipidemia, and endocrine factors, and so current in vitro protocols remain insufficient to fully reproduce the in vivo circumstances [9,10].

Animal models therefore remain essential for studying the in vivo pathomechanisms of restenosis and testing new pharmacological and device-based interventions. An ideal experimental model should be readily available, cost-effective, and ethically acceptable, while closely replicating human vascular anatomy, physiology, and disease progression [11]. Importantly, it should allow reproducible intimal hyperplasia and vascular remodeling induction in response to clinically relevant percutaneous interventions. Species-specific differences in vascular biology and healing responses significantly influence experimental outcomes [12].

In this review, we aim to summarize and critically evaluate the animal models used to study restenosis and intimal hyperplasia following percutaneous cardiovascular procedures, highlighting their advantages, limitations, and translational relevance.

## 2. Materials and Methods

### 2.1. Literature Search Strategy

A narrative literature review was performed to identify experimental studies investigating restenosis and intimal hyperplasia in animal models following percutaneous cardiovascular interventions. The databases PubMed, Embase, Web of Science Core Collection, and the Cochrane Library were searched using combinations of keywords related to restenosis, intimal hyperplasia, animal models, and percutaneous cardiovascular interventions.

This search was not intended to be exhaustive but rather to capture representative and influential studies that have contributed to understanding restenosis mechanisms and model development. The search covered studies published from 1990 to 2022, and no language restrictions were applied.

### 2.2. Study Selection and Scope

Relevant publications were selected based on their scientific relevance, methodological quality, and contributions to the field, and were included if they described in vivo animal models used to investigate vascular injury responses following percutaneous interventions, such as balloon angioplasty or stent implantation. A total of 46 were screened, of which 14 representative studies were included.

We did not consider in vitro studies, human clinical studies, and reports focusing exclusively on open surgical bypass procedures. Given the heterogeneity of animal species, vascular beds, experimental designs, and outcome measures, a narrative review was deemed the most appropriate. The selected literature was used to qualitatively synthesize commonly employed animal models, highlighting their advantages, limitations, and translational applicability, rather than performing a quantitative comparison.

## 3. Results

### 3.1. Study Selection and Scope (Table 1)

The studies discussed in this review were selected to represent commonly used and well-established experimental approaches that have contributed substantially to the current understanding of vascular injury responses and intimal formation.

The reviewed publications encompassed investigations focusing on coronary as well as peripheral arterial interventions and included both small- and large-animal models. Several studies specifically examined coronary interventions [13,14,15,16], while others explored peripheral arteries or compared multiple species to test devices, such as drug-eluting stents [17,18,19]. Rather than providing an exhaustive enumeration of all available studies, in this narrative review, we emphasize highlighting characteristic features of each animal model, including technical feasibility, biological response to injury, and translational relevance.

This qualitative synthesis allows for a comparison of vascular healing responses across species and facilitates discussion of how interspecies differences may influence experimental findings’ interpretations. By focusing on representative and influential studies, through this review, we aim to provide practical guidance for selecting appropriate animal models in preclinical restenosis research.

**Table 1:** Overview of included studies involving animal models of restenosis and intimal hyperplasia.

Study	Species	Purpose	Short Summary
Jeremy 2010 [11]	Mouse, Rat, Rabbit, Swine	Mechanistic and device evaluation	Time course of neointima formation characterized
Iqbal (2016) [13]	Mouse, Rat, Rabbit, Swine, Sheep, Dog	Device and drug testing	Compared stent types and neointimal responses across species
Taavitsainen (2020) [14]	Rabbit, Swine	Device evaluation	Neointimal proliferation evaluated post-stenting
De Prado (2013) [15]	Rabbit, Swine	Stent performance	Neointimal thickness quantified post-implantation
Suzuki (2009) [16]	Rabbit, Swine	Drug/device testing	Evaluated local drug effects on intimal hyperplasia
Bayes-Genis (2000) [17]	Mouse, Rat, Rabbit, Sheep	Mechanistic and stent testing	Evaluated vascular injury; interspecies differences noted
Perkins (2019) [18]	Swine	Device testing	Assessed long-term neointimal formation
Perkins (2010) [19]	Rat, Rabbit, Swine, Dog, Sheep, Nonhuman primates	Device evaluation	Compared DES vs. bare metal stents; species-dependent responses
Schwartz (2004) [20]	Mouse, Rat, Rabbit, Swine	Device evaluation	Studied responses to stent implantation
Ferns (2000) [21]	Mouse, Rabbit, Swine, Nonhuman primates	Neointimal formation	Characterized smooth muscle proliferation and remodeling
Touchard (2006) [22]	Rat, Mouse, Swine	Restenosis mechanism	Balloon injury + diet increased intimal hyperplasia
Amatruda (2014) [23]	Swine	Drug/device testing	Swine arteries mimicked human restenosis
Mitsutake (2017) [24]	Swine	Neointimal mechanisms	Documented SMC proliferation and vessel remodeling
Kantor (1999) [25]	Rat, Rabbit, Swine	Mechanistic	Balloon-injury-induced neointima; species-specific responses

### 3.2. Animal Models by Species (Table 2)

#### 3.2.1. Mouse Model [11,14,17,18,22,23]

The mouse has become a widely used experimental model in biomedical research; however, few mouse models are suitable for studying restenosis, mainly because the small size of mouse vessels limits dilatation and stenting procedures. The mouse aorta has a diameter of approximately 1 mm, presenting significant technical challenges for studies on intimal hyperplasia. In addition, there are important morphological differences compared with humans: mice lack the characteristic thick fibrous cap and vasa vasorum in atherosclerotic plaques, resulting in the absence of neoangiogenesis. These neo-vessels are important mediators of the inflammatory response in human vascular disease.

Despite these limitations, mice offer key advantages as experimental models: they are easy to handle and maintain, and they can be genetically manipulated, with various inbred strains and genetically modified mice available. For studying neointimal hyperplasia, the ApoE–deficient mouse with carotid artery ligation provides a model that partially mimics human pathology. ApoE-deficient mice have elevated cholesterol levels and develop atherosclerosis on a normal diet. Carotid artery ligation near the bifurcation disrupts blood flow, inducing neointima formation, vascular remodeling, and a reduction in vessel diameter in the common carotid artery within 2–4 weeks. Nevertheless, the absence of a robust inflammatory response and the small vessel size limit the clinical translatability of this model for studying neointimal behavior.

**Table 2:** Animal models used to study cardiovascular restenosis and intimal hyperplasia.

Species	Key Features	Main Advantages	Main Limitations	Typical Application	Translational Relevance
Mouse	~1 mm vessels, genetic manipulation	Low cost, powerful genetic tools	No clinical stenting, simple plaques	Mechanistic studies	Moderate
Rat	Larger vessels, surgical access	Reproducible injury models	Weak atherosclerosis	Neointima, drug testing	Moderate
Rabbit	Human-sized arteries, hyperlipidemia	Double-injury model	Elastic arteries	Restenosis, stents	High
Pig	Human-like coronary anatomy	Human-like neointimal response	High cost, rapid growth	Devices, PCI	Very high
Sheep	Stable physiology	Chronic implant suitability	Cost, handling	Valves, implants	High
Dog	Human-like conduction system	Electrophysiology relevance	Resistant to atherosclerosis	EP studies	Moderate
NHP	Closest to humans	Best physiological similarity	Ethics, cost	Safety validation	Very high

Abbreviations: PCI, percutaneous coronary intervention; EP, electrophysiology; NHP, nonhuman primates.

#### 3.2.2. Rat Model [11,14,15,17,19,20,23,24,25]

One of the oldest and most studied models is the rat, as it combines the low cost of mice with larger, more tractable vessels, although their vessels are still relatively small. Wild-type rats are largely resistant to diet-induced atherosclerosis, which has limited their use in this research. Endovascular procedures in rats often require specially designed stents or delivery systems, as well as advanced surgical skills. As with mice, transgenic, diabetic, obese, and hypertensive strain availability is an advantage; however, their vascular response to injury is similar to that of mice, showing minimal thrombus formation and inflammation—an important limitation for translational studies.

#### 3.2.3. Rabbit Model [11,14,15,17,18,19,20,21,22,25]

Rabbits are small, easy to handle, and relatively inexpensive. The sizes of their aortas, carotids, and iliofemoral arteries are comparable to human coronary arteries, making them suitable for evaluating commercially manufactured devices. As in the murine model, the degree of intimal hyperplasia, thrombus formation, and inflammatory response is lower in their more elastic-natured aorta/iliac arteries than in more muscular human arteries. Rabbits have been used extensively to study atherosclerosis and restenosis because they are susceptible to diet-induced hypercholesterolemia. Rabbit models are often referred to as “the double injury model”; the first lesion involves the development of atherosclerotic lesions. On a cholesterol-rich diet, rabbits develop atheromatous plaques rich in lipids with foam cells, which are similar to fatty streaks in humans. The second injury, usually balloon angioplasty, de-endothelializes the vessel and accelerates intimal hyperplasia, with cell infiltration beginning within days. The lesion character depends on dietary protocol, mechanical injury, and timing. Nonetheless, rabbit lesions differ histologically from human atheromas: they are foam-cell-rich and show less calcification, necrosis, and fibrosis. Long-term experiments using high-cholesterol diets are challenging because hepatotoxicity limits animal survival.

#### 3.2.4. Swine Model [11,14,15,16,17,18,19,20,21,22,24,25]

Of all animal species explored, the pig is the most similar to humans in its cardiovascular morphology and physiology. The anatomy of swine is like that of humans in regard to size, collateral arterial supply, and vasa vasorum presence. This model is suitable for evaluating stent and catheter performance in arteries analogous to diseased human arteries. The intimal response is of a similar histology to that in human restenotic coronary arteries. Major disadvantages of using such a large animal model include high housing costs, rapid growth, and high body weight; long-term studies are logistically difficult because of these factors, in addition to handling and equipment limitations. Swine do develop diet-induced atherosclerosis, but the added expense and time required for lesion development have limited the routine use of these models for restenosis or intimal hyperplasia studies.

#### 3.2.5. Other Large Animal Models: Dogs, Sheep, and Nonhuman Primates [14,19,22]

Large animal models generally offer good technical feasibility and reasonable translational relevance. The disadvantages include high costs, labor-intensive procedures, and ethical considerations.

Dogs: The dog model is not very useful to study atherosclerosis or restenosis; dogs are naturally resistant to atherosclerosis and intimal hyperplasia development. When atherosclerosis is induced via diet, dogs develop medial thickening and only a small neointimal layer, without significant lumen narrowing.

Sheep: Sheep are often overlooked as candidates for evaluating cardiovascular disease. Advantages of the sheep model include docile behavior and a coagulation and fibrinolytic system similar to humans. Disadvantages are similar to those in swine, including high housing costs and handling difficulties.

Nonhuman primates (NHPs): Due to their close physiological similarity to humans, monkeys could be a logical choice as animal models. However, ethical concerns; the need for specialized facilities, equipment, and enrichment; and high costs are major limitations. From a scientific perspective, NHPs provide excellent translational relevance: their development of atherosclerosis, lesion characteristics, and topography are highly similar to humans.

## 4. Discussion

Animal testing has played a central role in percutaneous coronary intervention development. Gruntzig performed the first coronary balloon angioplasty in the coronary arteries of dogs [26]. Restenosis, whether acute or chronic, soon emerged as a major problem, leading to coronary stent development, with Charles Dotter introducing the concept of stenting using canine models [27]. Porcine and rabbit iliac artery models were subsequently used to study restenosis, with therapeutic agents administered systemically or locally [28,29].

As Bernard Rollin stated, “The most brilliant design, the most elegant procedures, the purest reagents, along with investigator talent, public money, and animal life are all wasted if the choice of animal is incorrect” [30]. Understanding the biology of an intervention in animal models is essential to successfully translating results to humans [31]. Species differences—both in vessel size and structure—must be carefully considered. As Muller et al. noted [32], response to injury differs between muscular and elastic arteries, and so response to vascular injury varies substantially across species. Although neointima formation via smooth muscle cell migration, proliferation, and matrix synthesis is a common response, the volume and nature of neointima differ markedly. For example, in the rat carotid model, a thin platelet layer forms immediately after balloon withdrawal, whereas in the rabbit iliac model, macroscopic thrombus deposition occurs [33]. A fibrin-rich mural thrombus may serve as a nidus for medial smooth muscle cell colonization, as observed in pig and rabbit models, potentially explaining why some antiproliferative therapies that are effective in animal models fail to translate clinically [34].

Each animal model has unique strengths and limitations that should be fully exploited when evaluating therapies. For instance, rabbit and swine models are suitable for studying drug effects on smooth muscle cell proliferation, but results should not be directly extrapolated to humans; direct translation from animals to humans is unreasonable, given interspecies differences and restenosis complexity. Translational success also depends on methodological alignment. Clinical studies commonly rely on quantitative angiography, while animal studies rely on histopathology, which is more sensitive and may detect smaller changes in neointima formation. Additional factors explaining discrepancies include differences in drug dosage, small sample sizes in clinical trials, and variations in study endpoints [35].

Careful consideration of interspecies similarities and differences is essential for designing experiments that can effectively translate bench findings to clinical practice, in alignment with the 3Rs principle (Replacement, Reduction, and Refinement). Current restenosis research applies the 3Rs principle by refining procedures to reduce animal suffering, minimizing animal numbers through longitudinal designs, and partially replacing in vivo experiments with advanced in vitro and computational models, thereby enhancing ethical compliance while maintaining translational relevance.

Restenosis remains clinically relevant despite modern percutaneous interventions [36]. In humans, it is a complex, multifactorial process driven by endothelial injury, platelet activation, inflammatory cell recruitment, SMC proliferation, and extracellular matrix deposition, processes that occur within a biomechanically and metabolically altered vascular environment, often characterized by advanced atherosclerosis, disturbed shear stress, and systemic comorbidities such as diabetes and dyslipidemia. Animal models differ substantially in their ability to reproduce these interacting pathophysiological mechanisms, which directly affects their translational relevance. Small-animal models, particularly mice and rats, are well suited for dissecting the molecular and genetic pathways involved in neointimal formation, such as inflammatory signaling, SMC proliferation, and endothelial regeneration. However, their limited thrombotic response, complex plaque architecture absence, and small vessel size mean that they only partially reflect human restenosis, which is strongly influenced by inflammation–thrombosis interactions and plaque burden. These models therefore primarily inform mechanistic understanding rather than direct clinical translation.

In contrast, rabbit and swine models more closely approximate key human vascular characteristics, including vessel size, flow patterns, and vasa vasorum presence. In particular, porcine models reproduce the human-like neointimal response to stent implantation, including SMC-driven hyperplasia and extracellular matrix accumulation, making them highly relevant for evaluating device-based therapies. Importantly, the fibrin-rich thrombus formation observed in larger animal models more closely resembles the early human response to vascular injury.

From a practical and translational perspective, animal model choice should be driven by the specific research question. Mechanistic studies aimed at identifying molecular targets may appropriately rely on genetically modified murine models, whereas preclinical evaluation of stents, drug-coated balloons, or local drug delivery systems requires large-animal models that replicate human vascular dimensions and healing responses. Failure to align the experimental model with the intended clinical application risks misleading conclusions and unsuccessful translation.

Recent methodological advances may further refine preclinical restenosis research without replacing established in vivo models. Hybrid in vivo–in silico approaches integrating animal data with computational modeling offer the potential to improve vascular healing prediction and reduce experimental variability. In addition, imaging-guided endpoints, such as intravascular ultrasound or optical coherence tomography, together with molecular phenotyping approaches, may enhance mechanistic insight and translational relevance. At present, these strategies are best viewed as complementary tools that support model selection and interpretation rather than as standalone alternatives.

Computational and artificial-intelligence-based models can help to predict vascular responses, optimize experimental design, and reduce reliance on animal experiments. Hybrid approaches, combining in vitro and small-animal studies, may further enhance mechanistic insights while reducing the number of large animals needed. Animal models may no longer serve as primarily discovery tools but rather as confirmatory and integrative components within computational research frameworks.

Taken together, animal models should not be viewed as direct surrogates for human disease but as complementary tools within a translational research pipeline. Their value lies in strategically matching biological complexity to the study objective, thereby maximizing clinical relevance while adhering to ethical principles. Strengthening this alignment is essential for improving the predictive value of preclinical restenosis research and guiding effective cardiovascular intervention development.

This narrative review has several limitations: It was not conducted as a systematic review and therefore lacks a predefined protocol, formal quality assessment, and quantitative synthesis, which may introduce selection bias and limit the completeness of the included literature. The available studies are highly heterogeneous with respect to animal species, vascular beds, injury models, and outcome measures, which restricts direct comparison and precludes definitive conclusions regarding the optimal model for restenosis research. Finally, in this review, we focus on in vivo animal models and do not incorporate emerging alternatives such as advanced in vitro or computational approaches. Despite these limitations, we provide a focused overview of established animal models and highlight key considerations for their appropriate use in translational restenosis research.

## 5. Conclusions

Animal models are important for understanding restenosis and neointimal hyperplasia and remain essential for testing new treatment modalities. The ideal experimental model should reliably predict clinical trial outcomes; however, differences in injury severity as well as interspecies variations in healing responses and metabolism make it challenging to draw direct conclusions about humans based on animal data.

While it is important to recognize these limitations and the differences between animal models and the human condition, such models have nonetheless provided valuable insights into the mechanisms of restenosis following cardiovascular interventions, with each model having its own strengths and weaknesses. Given the persistently high restenosis rate despite technological advances, further research into its pathophysiological mechanisms is needed. Careful selection of the most appropriate animal model is therefore crucial for the success of translational research.

Animal model use will remain subject to strict ethical and regulatory oversight.

In summary, the role of these models is shifting from broad discovery to targeted, high-value validation in the cardiovascular innovation pipeline, complemented by alternative and computational approaches to maximize translational relevance and ethical compliance.

## Data Availability

All data presented in this study are included in the references cited in the manuscript. No new data were generated in this study.

## Abbreviations

The following abbreviations are used in this manuscript:

PCI	Percutaneous Coronary Intervention
DES	Drug-Eluting Stent
NHP	Nonhuman Primate
SMC	Smooth Muscle Cell
ApoE	Apolipoprotein E
EP	Electrophysiology
3Rs	Replacement, Reduction, Refinement
